# Strong Coupling between a Quasi-single Molecule and a Plasmonic Cavity in the Trapping System

**DOI:** 10.1186/s11671-019-2886-1

**Published:** 2019-03-01

**Authors:** Yunfei Zou, Gang Song, Rongzhen Jiao, Gaoyan Duan, Li Yu

**Affiliations:** 1 State Key Laboratory of Information Photonics and Optical Communications, Beijing University of Posts and Telecommunications, Xitu Cheng Road, Beijing, 100876 China; 2 School of Science, Beijing University of Posts and Telecommunications, Xitu Cheng Road, Beijing, 100876 China

**Keywords:** Strong coupling, Plasmonic, Blue-detuned trapping

## Abstract

We theoretically investigate the strong coupling phenomenon between a quasi-single molecule and a plasmonic cavity based on the blue-detuned trapping system. The trapping system is made up of a metallic nanohole array. A finite-difference time-domain method is employed to simulate the system, and the molecule is treated as a dipole in simulations. By calculating the electromagnetic field distributions, we obtain the best position for trapping a molecule, and we get the strong coupling phenomenon that there are two splitting peaks in the transmission spectrum when the molecule is trapped in the structure, while only one peak is observed in the one without the molecule. We also find that only when the molecule polarization parallels to the incident light wave vector can we observe a strong coupling phenomenon.

## Introduction

In recent years, using optical dipole traps to trap and cool atoms or molecules has been a promising technology to achieve the Bose-Einstein condensate, test fundamental physical laws, and measure basic physical constants more precisely [1–3]. Optical dipole traps primarily use the gradient forces of the incident light to produce a dipole effect on an atom. For the red-detuned traps, the atoms are trapped in the position where the light intensity is strongest under attractive potential [4]. Owing to the Rayleigh and Raman scattering, the trapped atoms will be subjected to apparent atomic coherence and heating effect. At the same time, in the strongest light position, the nuclear energy level has a severe optical frequency shift [1]. On the contrary, for the blue-detuned traps [5, 6], the atoms are trapped in the area of weakest light intensity under exclusion potential [6–8]. Compared with red-detuned traps, the scattering rate of a photon can be significantly reduced with a weak intensity of incident light, and so, that provides a more stable way to trap the small particles. However, the construction of the blue-detuned traps is often complex [9, 10]. Surface plasmon polaritons (SPPs) are hybrid modes of light waves coupled to free electron oscillations confined at the interface between a dielectric and a metal, which have tremendous potential for a wide range of applications in the field of THz devices [11–14], materials [15], sensors [16], meta-surface [17], and quantum information processing [18]. Combining optical trapping with plasmonic structures is a possibility to develop integrated optics components. For example, Chang et al. proposed using a nanostructure with a combined nanotip and microdisk cavity for isolated atom trapping [19]; Chen et al. achieved stable 3D atom trapping based on blue-detuned light in an array of plasmonic nanoholes [20]. A periodic plasmonic nanostructure with an array of subwavelength holes has interesting optical property, which is an encouraging scenario to trap a small particle such as an atom or a molecule.

Strong coupling between a plasmonic cavity and a molecule not only attracts interest in the fundamental study of quantum electrodynamic phenomenon, but also has a bright prospect in quantum information processing [21–23]. Plasmonic cavities, which can significantly enhance the strength of the light-matter interaction, are an appropriate candidate to achieve the strong coupling at room temperature [24–26]. However, a single molecule coupling with a plasmonic cavity is a huge challenge in both theory and experiment, which is difficult to manipulate the position of the molecule in the coupling system.

In this paper, we theoretically study the strong coupling between a quasi-single molecule and a plasmonic cavity for each unit of a blue-detuned trapping system. The blue-detuned trapping system is made up of a gold nanohole array, and each unit only traps one molecule. Finite-difference time-domain (FDTD) method is employed to simulate our structure, and the transmission spectrum can be obtained. When the molecule is trapped at the weakest point, the scattering spectrum exhibits Rabi splitting, a signature of strong coupling regime. The proposed structure provides a potential way to achieve the strong coupling between a quasi-single molecule and a plasmonic cavity in the optical trapping system.

## Methods

We design a periodic nanohole structure to achieve the blue-detuned trapping. Our simulations are based on FDTD methods, and we adopt the EAST FDTD software to set our structure and investigate the transmission spectrum and the electromagnetic distribution. In our model, a series of gold nanohole units with a radius R are arranged in a two-dimension (2D) Au film in the *X*-*Y* plane and the thickness of the Au film is 400 nm. The lattice constant *L* and the radius *R* are 1000 nm and 250 nm, respectively. During our simulation, the background index is 1 and the gird is 5 nm for each direction. The periodic boundary conditions are set along both the *X*-axis and *Y*-axis. The perfect match layer is set along the *Z*-axis. The number of the perfect match layer is 32. The dielectric constant of Au is obtained from Johnson and Christy [27]. Circular polarization light normally injects onto the surface of the proposed structure along the *Z*-axis, and the wavelength is 696 nm. An *X*-*Y* plane recorder is 400 nm away from the proposed structure surface to calculate the transmission and an *X*-*Z* plane recorder is in the center of the structure to get the electromagnetic field distribution. Owing to the property of the dipole source in FDTD simulation [28–30], we can use the dipole source to simulate the molecule. The resonant wavelength of the molecule is 707 nm, and the decay rate is 1.1×10^14^ Hz. In our simulation, the transmission *T* is calculated by integrating the Poynting vector over the upper surface and normalizing to that obtained in the absence of the metal structure [31]. The transmission calculated in FDTD simulation is denoted as *T*=*I*_*T*_/(*I*_*C*_+*I*_*D*_), where *I*_*T*_ is the electromagnetic field intensity of the transmission, *I*_*C*_ is the electromagnetic field intensity of circular polarization light, and *I*_*D*_ is the electromagnetic field intensity of the dipole.

## Results and Discussion

### Trapping Structure

**Fig. 1:**
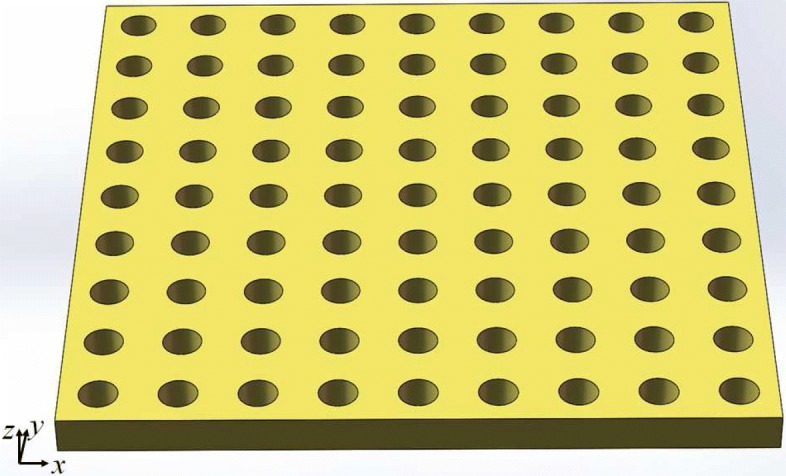
Schematic diagram of the periodic plasmonic nanostructure

Plasmonic nanostructure with an array of subwavelength holes exhibits an extraordinary optical transmission effect which will considerably enhance the local electric field near the nanoholes [32–34]. We use this effect on molecular motions. When a plasmon resonant field is blue-detuned from the molecular resonance, a giant repulsive force of the molecule can be produced and a trapping minimum about hundreds of nanometers away from the structure surface will be created. We design a periodic plasmonic nanostructure to demonstrate our approach, as shown in Fig. 1.

**Fig. 2:**
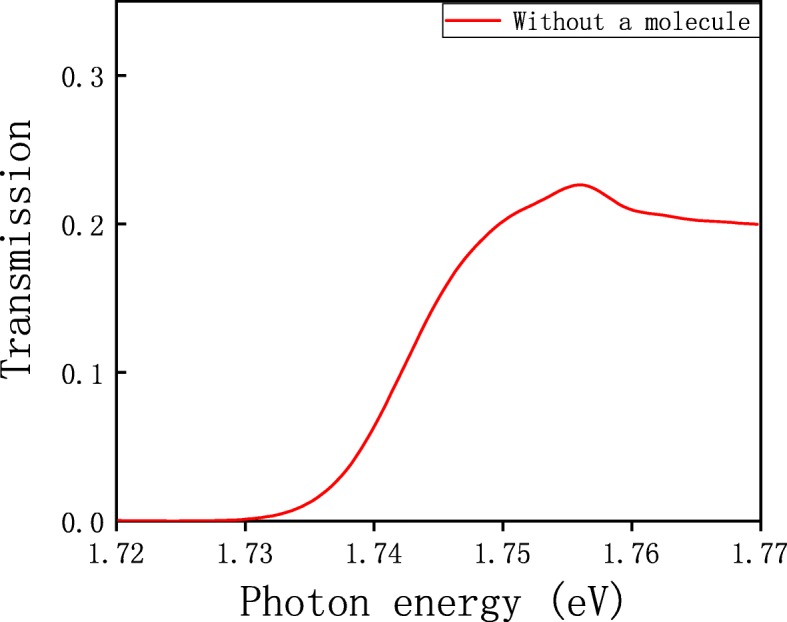
The transmission spectrum of the structure without a molecule

In this section, we only discuss the simulation results without a molecule. We use a circular polarization light to normally illuminate the structure from the *Z* direction at infinity, and the transmission spectrum is displayed in Fig. 2.

**Fig. 3:**
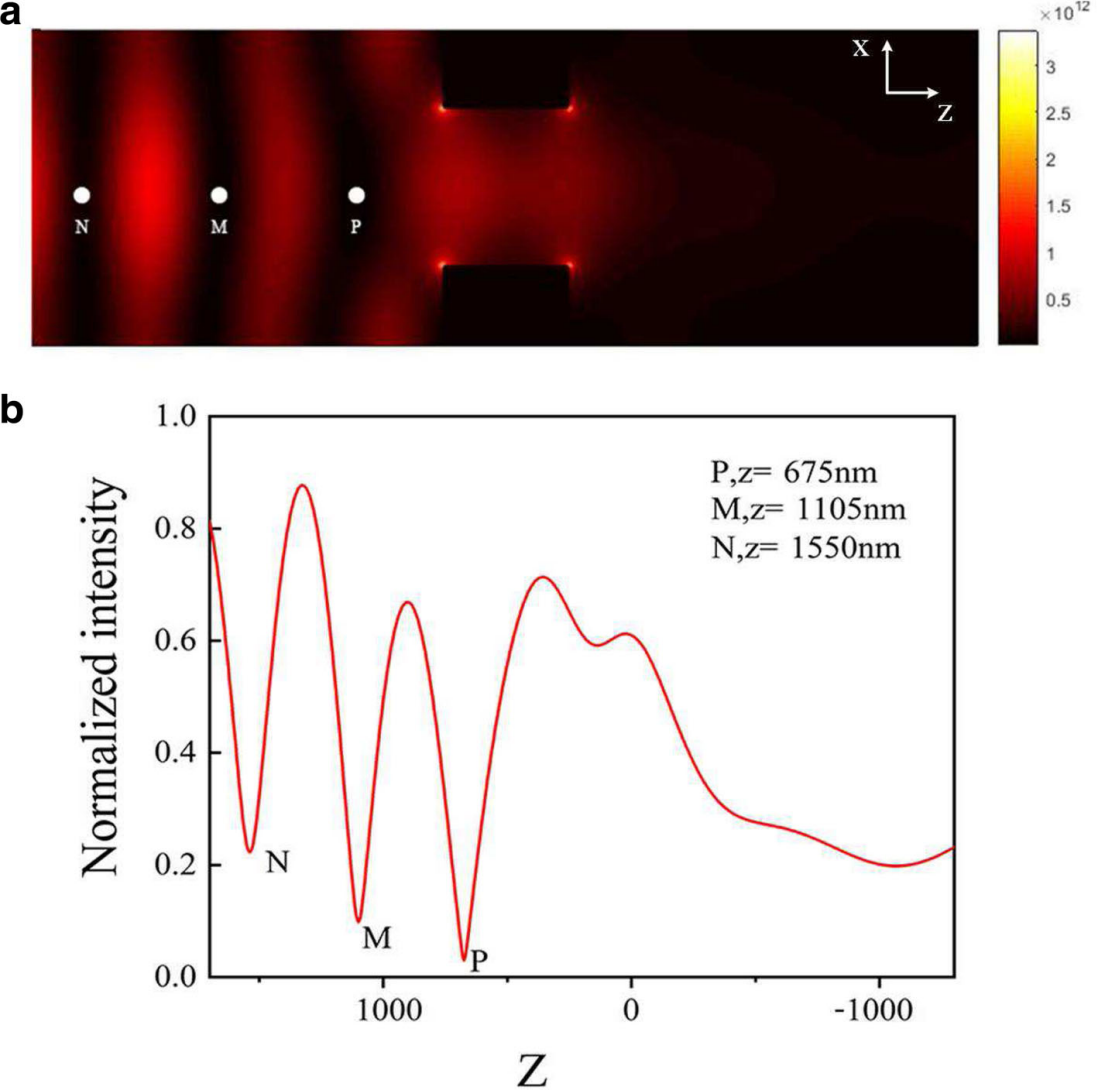
**a** Electromagnetic field intensity |*E*|^2^ distribution in the *X*-*Z* plane at *Y* = 0 at *λ*=696 nm. **b** Normalized intensity distributions of |*E*|^2^ versus *Z*with *X* = 0 and *Y* = 0 in the *X*-*Z* plane

A resonant peak is observed at 707 nm (1.756 eV), which originates from the interaction among units (the holes). This peak is close to the wavelength of (1, 1) order of Wood anomaly [35]. An array of subwavelength holes has Bragg scattering to the SPPs, bringing about a reciprocal lattice vector of the array which can easily satisfy the phase-matching condition [20]. Thus, incident light can excite the SPPs, and this way of exciting SPPs is much easier than using the Kretschmann structure and is not influenced by the thickness of the metal. For the purpose of investigating the spatial electromagnetic field distribution of the system, we plot a figure of |*E*|^2^ distribution of the *X*-*Z* plane at *Y* = 0 at the resonant wavelength of 707 nm, which is shown in Fig. 3a.

In Fig. 3a, there are three minima of |*E*|^2^ noted by the white points P, M, and N; the appearance of these three minima can be attributed to the hot spots arising, which result from the near-field scattering of light by an array of plasmonic nanoholes. Actually, the distribution of electromagnetic field is the superposition of the surface plasmon field and the spatial electromagnetic field through the nanoholes. The curve of |*E*|^2^ versus *Z* at *X* = 0 and *Y* = 0 in the *X*-*Z* plane is also plotted in Fig. 3b. According to the simulation result of the spatial electromagnetic distribution, there will be three intensity minima, which implies the blue-detuned optical trap.

A molecule can be trapped in electromagnetic field intensity minima via optical dipole forces with blue-detuned light. In our simulation, the molecule is treated as a two-level system and can be simulated as a dipole in FDTD simulation [28–30]. The trapping potential *U*_opt_ for a molecule is a repulsive optical dipole potential associated with electromagnetic field *E*, which is given by [1, 4]:

1 $$ U_{\text{opt}} = - 0.25\alpha {\left| E \right|^{2}}  $$

**Fig. 4:**
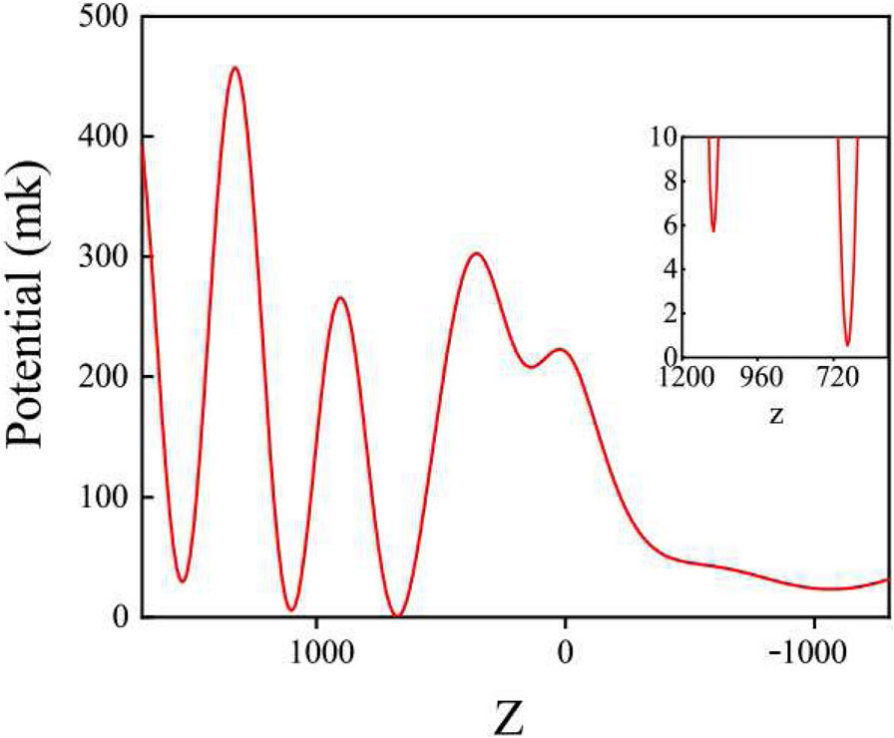
Trapping potential in *X*-*Z* plane at *Y* = 0. The insert picture is the scale-up of the lowest trapping potential

Here, *α* is reduced polarizability, in our case *α*=−7.87×10^−38^*F*·*m*^2^ [20]. Therefore, on the basis of the electromagnetic field intensity distributions, we can obtain the trapping potentials. Using Eq. (1) and |*E*|^2^ distributions in Fig. 3a, we calculated the trapping potential along the line with both *X* = 0 and *Y* = 0 as displayed in Fig. 4.

**Fig. 5:**
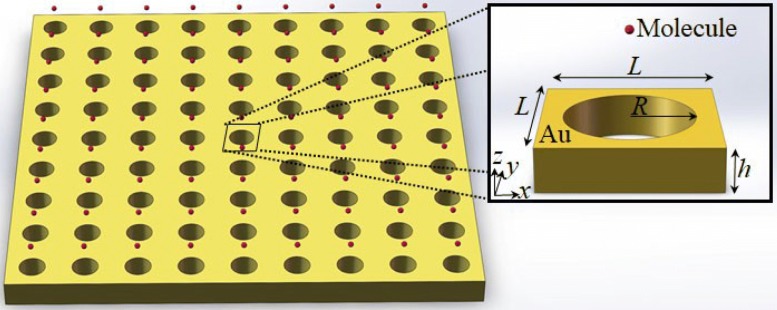
Schematic diagram of a molecule trapped in the structure

In Fig. 4, we know that the lowest intensity position is at point P. Here, the incident power is set as *P*_*i*_=120 mW, and the trapping potential is 0.53 mK. Chen et al. has reported that ^87^*Rb* can be trapped stably with the trapping potential of 2.02 mK [20]. Comparing these parameters, our blue-detuned trapping system could trap a rhodamine molecule with the molecular weight about 400. In the light of the analysis above, we choose the trapping position at the point (0, 0, 675 nm), and the schematic diagram of a molecule trapped in the structure is shown in Fig. 5.

### Strong Coupling Between Structure and a Molecule

**Fig. 6:**
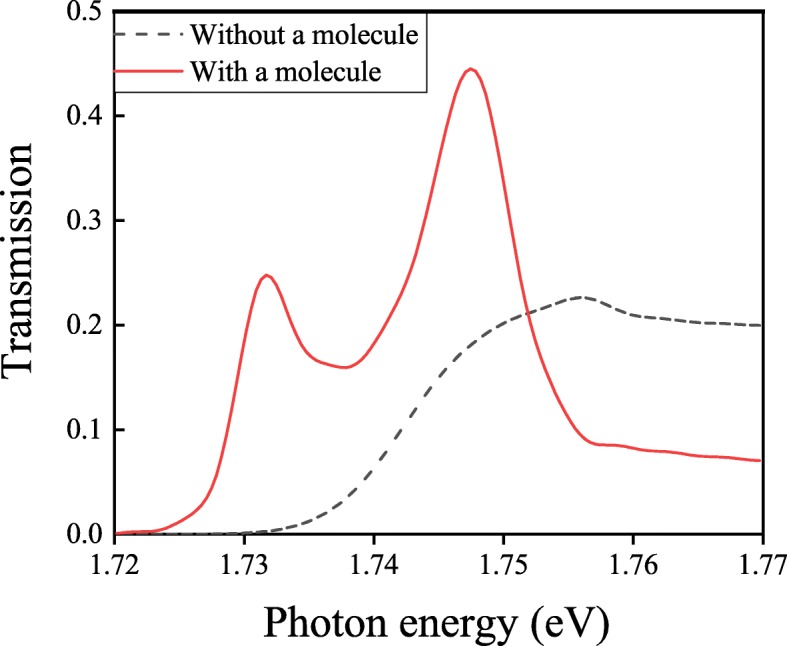
Transmission spectra with and without a molecule is trapped at point P

Molecules are placed into each unit of our structure to study the light-molecule interaction at strong coupling regime. The molecule can be treated as a dipole in FDTD simulations. We add a dipole at the potential minimum point P, which is 275 nm away from the surface, as shown in Fig. 3a. The polarization of the dipole could be along the *X*-axis, the *Y*-axis, or the *Z*-axis. The resonant wavelength of the molecule is 707 nm (1.756 eV). First, we consider the polarization of the dipole along the *Z*-axis. The transmission spectrum is also obtained and the result is in Fig. 6.

**Fig. 7:**
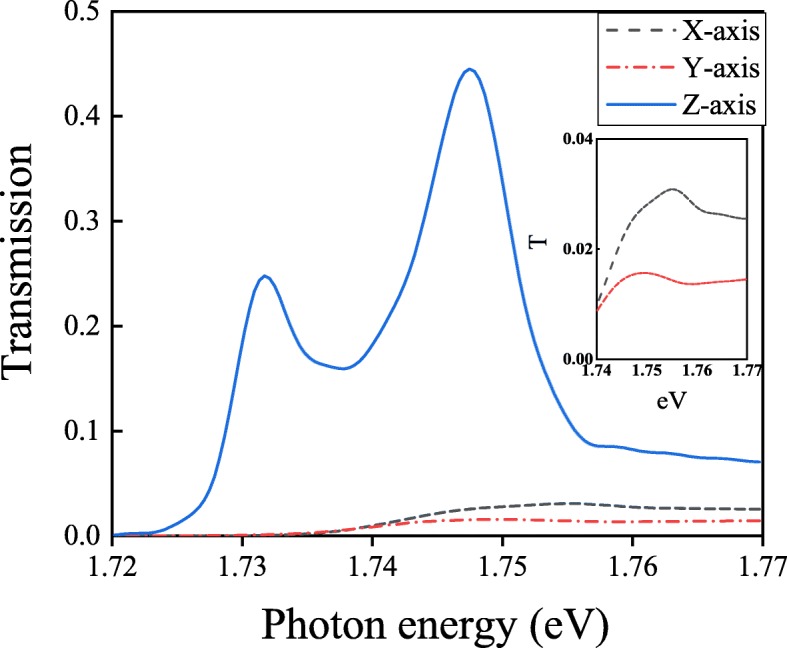
Transmission spectra with a molecule polarized along the X, Y, and Z directions, respectively

For comparison, the transmission spectrum of the structure without a molecule in each unit is also plotted in Fig. 6. In line with Fig. 6, we can observe two splitting peaks in the transmission spectrum of the structure with a molecule, which shows the Rabi splitting lineshape and presents the strong coupling phenomenon. Incident light not only injects onto the surface of our structure but also makes the molecule excited. SPPs excited by incident light couple with the ones excited by the molecule around the working wavelength. These two kinds of SPPs couple with each other and make two splitting peaks in the transmission spectrum. The lineshape of the transmission curve shows the strong coupling phenomenon. In generic, the frequencies of these two splitting peaks *ω*_±_ are denoted as [36, 37]:

2 $$ \omega_{\pm} = \omega_{0} - 0.25i(\gamma_{c}+\gamma_{m}) \pm \sqrt{[g^{2}-0.25(\gamma_{c}-\gamma_{m})^{2}]}  $$

where *ω*_0_ is the energy under the condition that the isolated molecule and cavity are assumed to be in resonance, *γ*_*c*_=4.08×10^13^Hz [38] and *γ*_*m*_=1.1×10^14^Hz are the decay rates of the plasmonic cavity and the molecule, respectively, and *g* is the coupling constant. Strong coupling occurs for *g*>0.5|*γ*_*c*_−*γ*_*m*_| and corresponds to the formation of a dressed state with a finite lifetime. For our proposed structure, the coupling constant *g* is 144 meV, while 0.5|*γ*_*c*_−*γ*_*m*_| is 143 meV. In previous works, Rabi splitting denoted as *Ω*=|*ω*_+_−*ω*_−_| is in the regime from 100 to 450 meV in the structures based on the J aggregate-metal or the molecule-metal hybrid nanostructures [39, 40]. The Rabi splitting is related to the coupling strength *g*, which depends on$\sqrt {N/V}$, where *N* is the number of molecules and *V* is mode volume, respectively [37]. In our work, there is only one molecule in each unit of the proposed structure and the mode volume *V* is so large that the Rabi splitting *Ω* is about 16 meV, this splitting is comparable with strong coupling in metal-semiconductor hybrid nanostructures [41] and metal-2D material system [42]. We also have studied the strong coupling of the structure with the polarization of the molecule along the *X*-axis and the *Y*-axis, respectively. The simulation results are displayed in Fig. 7.

In Fig. 7, two splitting peaks (710 nm, 1.747 eV; 717 nm, 1.717 eV) only appear in the structure with the polarization of the molecule along the *Z*-axis. The molecule polarized along the *X*-axis or the *Y*-axis shows a strong collective behavior before the coupling with Au nanocavity or Au nanohole array. The collective behavior *S*_*k*_ of the molecule polarized along the *X*-axis or the *Y*-axis can be calculated by the coupled-dipole method [43]:

3 $$ {S_{k}} = \sum\limits_{\text{dipoles}} {{e^{i{k_{0}}r}}\left[ {\frac{{\left({1 - i{k_{0}}r} \right)\left({3{{\cos }^{2}}\theta - 1} \right)}}{{{r^{3}}}} + \frac{{k_{0}^{2}{{\sin }^{2}}\theta }}{r}} \right]}  $$

where *k*_0_ is the wave vector in the vacuum, *θ* is the angle between two dipoles, and *r*=*nL*, *n* = 1, 2, 3... Here, with the condition of $ \lambda = \frac {L}{\sqrt {i^{2}+j^{2}}}$ (*i*, *j*= 0, 1, 2,..., but both *i* and *j* are not equal to 0 at the same time), the collective behavior is quite large, which presents a singularity in Eq. (3) [35, 43]. It exhibits a resonant dip in the transmission spectrum. For the molecule polarized along the *X*-axis or the *Y*-axis, the collective behavior is too large to weaken the coupling between the molecule and the plasmonic cavity. We only see the order of Wood anomaly [35] with the order of (1, 1) in the transmission spectrum around the wavelength 707 nm (1.756 eV), the strong coupling could not appear. Hence, only the molecule polarized along the *Z*-axis in our proposed structure can generate the strong coupling phenomenon. If the resonant wavelength of the molecule is changed, the strong coupling in the blue-detuned trapping system can also be obtained by the steps below. Firstly, according to the resonant of the molecule, the lattice constant of the nanohole array can be fixed. Secondly, the working wavelength of the blue-detuned trapping system can also be determined, which depends on the lattice constant of the metallic nanohole array. Thirdly, the electromagnetic field distribution is obtained to find the trapping position. Finally, we put the molecule at the best trapping position to calculate the transmission spectrum and the transmission with the strong coupling lineshape can be obtained.

## Conclusion

In summary, we design a metallic plasmonic blue-detuned trapping system and investigate the strong coupling phenomenon between a quasi-single molecule and the trapping system. In FDTD simulations, we use a dipole source as the molecule, and according to the electromagnetic field distributions, we can find the best position for trapping only one single molecule in the structure. By calculating the transmission of the proposed structure when the molecule has been trapped or not, we find that there are two splitting peaks in the transmission spectrum when the molecule is trapped in the structure, while only one peak is observed when the molecule is not trapped. The two splitting peaks indicate the strong coupling has happened. Hence, we can design the blue-detuned trapping system to achieve strong coupling in a nanoplasmonic structure, which has potential applications in quantum information processing.

## Abbreviations

FDTD: Finite-difference time-domain
